# Histological changes in HCV antibody–positive, HCV RNA–negative subjects suggest persistent virus infection

**DOI:** 10.1002/hep.22484

**Published:** 2008-12

**Authors:** Matthew Hoare, William T H Gelson, Simon M Rushbrook, Martin D Curran, Tracy Woodall, Nicholas Coleman, Susan E Davies, Graeme J M Alexander

**Affiliations:** 1 Department of Medicine, School of Clinical Medicine, University of CambridgeCambridge, UK; 2 Clinical Microbiology and Public Health Laboratory, Health Protection Agency, Addenbrooke's HospitalCambridge, UK; 3 Medical Research Council (MRC) Cancer Cell Unit, Hutchison/MRC Research CentreCambridge, UK; 4 Department of Pathology, University of CambridgeCambridge, UK

## Abstract

It is unclear whether hepatitis C virus (HCV) has been eradicated or persists at a low level in HCV antibody–positive HCV RNA–negative individuals. The natural history and liver histology are not well characterized. One hundred seventy-two HCV antibody–positive, serum HCV RNA–negative patients underwent diagnostic liver biopsy between 1992 and 2000 and were followed a median 7 years (range, 5–12). Patients with any possible cause of liver injury other than HCV were excluded. A single histopathologist scored sections using Ishak criteria. Characterization of the inflammatory infiltrate in selected cases used a novel semiquantitative technique and compared with HCV RNA–positive patients and healthy controls. One hundred two patients were excluded because of a risk factor for liver injury other than HCV. Seventy patients met the study criteria; four (5.7%) became HCV RNA–positive during follow-up. Sixty-six cases remained HCV RNA–negative; five (7.5%) had a normal liver biopsy; 54 (82%) had fibrosis (stage 2 or 3 in 16 (24%)). Nonviremic cases revealed expanded portal tracts (*P* < 0.05), with fewer CD4+ (*P* < 0.05) and more CD8+ cells (*P* < 0.05) than healthy controls, but were indistinguishable from HCV RNA–positive cases for these parameters. Lobular CD4 staining, absent in healthy controls, was noted in both HCV RNA–negative and –positive cases and was more marked in the latter (*P* < 0.05) with a sinusoidal lining cell distribution. *Conclusion:* Nonviremic HCV antibody–positive patients have a liver biopsy that is usually abnormal. Fibrosis was present in most with similar inflammatory infiltrate to viremic cases. The presence of a CD8+ rich inflammatory infiltrate suggests an ongoing immune response in the liver, supporting the view that HCV may persist in the liver in the majority of HCV RNA–negative cases. (Hepatology 2008;48;1737-1745.)

Hepatitis C virus (HCV) infection has a prevalence of 0.5%–2% in Western countries, with sustained viremia in 50%–90% of exposed individuals.^1^ Between 5% and 20% of those with viremia develop cirrhosis eventually^2^,^3^ and are then at risk of chronic hepatic failure and hepatocellular carcinoma. The gold standard for investigation of HCV-related disease remains liver biopsy. Sequential liver biopsies demonstrate progressive liver fibrosis in more than 50% of subjects with chronic viremia.^3^–^5^ Some studies have described the association of strong peripheral T cell responses with resolution of viremia immediately after acute HCV infection,^6^–^8^ which contrasts with the weak, narrow T cell response in viremic HCV carriers.^9^,^10^ There have been fewer studies of the intrahepatic lymphocyte compartment in individuals long after spontaneous resolution of viremia. There has been resurgent interest in this particular group following the demonstration of intrahepatic negative strand HCV RNA, suggesting continued viral replication,^11^ leading to the suggestion that such patients have occult or, alternatively, low-level HCV replication,^12^ but the effect of immune responses on viral turnover is uncertain.

The natural history of HCV-infected patients without viremia is believed to be excellent but is less well characterized, and histological abnormalities have been described in only a limited number of studies.^13^ A proportion of nonviremic HCV subjects continue to be identified in screening programs, but at present their optimal management remains undefined. Until 2000, the practice in our center was to offer full clinical assessment including liver biopsy, due to uncertainty of the natural history of nonviremic subjects.

In this series, the liver biopsy features in a cohort of HCV antibody–positive, HCV RNA–negative patients followed in a single center for at least 5 years are described. Other causes of liver injury had been excluded carefully, and the recognition that hepatic inflammation was a common feature in such patients led to further study to characterize the infiltrate in a subset of cases. Using immunohistochemistry, we compared the inflammatory infiltrate in a subset of HCV antibody–positive, viremic, and nonviremic subjects and healthy controls.

## Patients and Methods

We conducted a retrospective analysis of patients known to remain HCV antibody–positive but HCV RNA–negative (nonviremic) persistently that had undergone percutaneous liver biopsy in our center between July 1992 and December 2000. During this period, all patients who were anti-HCV antibody–positive were offered liver biopsy irrespective of RNA status.

Case inclusion was defined strictly to ensure that exposure to HCV was the only recognized cause of liver injury. All were HCV RNA–negative at presentation, and none had undergone therapy with interferon. Patients that consumed more than the recommended amount of alcohol per week (>21 U/week in men, >14 U/week in females) were excluded. Patients infected with human immunodeficiency virus (HIV) or hepatitis B virus (HBV) and those with other recognized causes of chronic liver disease identified on blood tests or liver biopsy were also excluded. Thus, all had a body mass index <30 without risk factors for insulin resistance; were negative for antimitochondrial, antinuclear, and anti–smooth muscle antibodies with normal serum immunoglobulins; had no evidence of iron overload; and had normal serum α1-antitrypsin, copper, and ceruloplasmin levels. Patients were analyzed according to age, sex, and risk factors for acquisition (Table 1).

**Table 1:** The Demographic Characteristics of 66 Nonviremic, HCV Antibody–Positive Patients

Nonviremic, HCV Antibody–Positive (n = 66)	Mean ± SD	Range
Age (years)	37.6 ± 8.6	21.2–65.7
Male/female ratio (%)	41:25 (62%:38%)	
Follow-up (years)	7	5–12
Number of HCV RNA assays per patient	7	5–12
ALT median (normal range) IU/L	31 (<40)	IQR, 22.25–38.75; range, 8–213
Lobular activity (0–4)	0.82 ± 0.65	0–2
Portal activity (0–4)	0.66 ± 0.60	0–2
Fibrosis (0–6)	1.1 ± 0.73	0–3
Interface activity (0–4)	0.11 ± 0.30	0–1
Confluent necrosis (0–6)	0.05 ± 0.20	0–1
Steatosis (0–3)	0.27 ± 0.57	0–3

ALT, alanine aminotransferase; IQR, interquartile range.

All study patients were followed for a minimum of 5 years (median, 7 years [range, 5–12]) with annual clinical assessment supported by laboratory tests including liver function tests, HCV antibody, and HCV RNA.

The study was performed with the approval of the local research ethics committee.

### HCV Antibody and Polymerase Chain Reaction for HCV RNA

Immunoglobulin G anti-HCV antibody was sought using the ADVIA Centaur sandwich immunoassay (Bayer, Newbury, UK). Prior to 2003, a nested blocked based reverse-transcription polymerase chain reaction (PCR) assay was used to detect HCV RNA. After 2003, HCV RNA was sought using a real-time Taqman PCR assay, targeting the conserved 5′ noncoding region of the HCV genome and performed on a Rotor-gene 3000 instrument (Corbett Lifescience, Sydney, Australia). Probit analysis (Stats Direct, www.statsdirect.com) revealed a limit of detection of 25 IU/mL (95% confidence interval, 6.3–38.6). The detection limit of the nested reverse-transcription PCR assay was not significantly different from the later real-time assay (data not shown). Patients were only included in this study if a minimum of 5 (maximum of 12) separate tests at 12-month intervals had failed to detect HCV RNA.

### Routine Liver Histology

Liver biopsies were performed with a 1.9-mm diameter Menghini needle. Biopsy specimens were fixed in 4% neutral buffered formaldehyde and embedded in paraffin. Four-micrometer sections were stained with Meyer's hematoxylin-eosin, periodic acid–Schiff with diastase pretreatment, Prussian Blue, a trichrome stain (van Gieson or chromotrope alanine blue), and Gomori's reticulin stain. All biopsies were examined by a single liver histopathologist (S. E. D.). Biopsies were classified according to modified Ishak criteria^14^ after assessing the adequacy of the specimen. Histological activity index represented the sum of interface hepatitis (0–4), confluent necrosis (0–6), lobular inflammation (0–4), and portal inflammation (0–4). Fibrosis was scored 0 (absent) to 6 (cirrhosis), and steatosis was scored 0–3. Features of steatohepatitis were recorded.

To further characterize the inflammation that was demonstrated at routine histology, the inflammatory infiltrate was investigated by immunohistochemistry in a subgroup of cases. A group of 12 nonviremic patients selected randomly from the original cohort with portal or lobular inflammation between Ishak 1 and 3, was compared with a group of 13 viremic patients and 18 controls. Liver tissue from viremic HCV patients (n = 13) was matched carefully for age, fibrosis stage, and inflammation grade with the nonviremic patients; these patients also met the strict entry criteria for the study group, except for the presence of HCV RNA in serum and served as a comparison group. The age and biopsy features of the two groups were the same except for increased interface activity in the viremic cohort (Table 2).

**Table 2:** Demographic Characteristics of Subjects Studied via Immunohistochemistry

	HCV Antibody and HCV RNA–Positive (n = 13)	HCV Antibody and HCV RNA–Negative (n = 12)	Healthy Controls (n = 18)	Statistic	*P* Value
Age (years ± SD)	35.59 ± 11.75	38.83 ± 7.99	48.46 ± 15.70	Kruskal-Wallis test	0.06
Lobular activity (0–4)	2.08 ± 0.29	1.75 ± 0.62	—	Mann–Whitney U test	0.11
Portal activity (0–4)	2.00 ± 0.60	1.83 ± 0.58	—	″	0.49
Fibrosis (0–6)	1.75 ± 0.75	1.92 ± 1.24	—	″	0.69
Interface hepatitis (0–4)	1.50 ± 0.5	0.83 ± 0.72	—	″	0.03
Steatosis (0–3)	1.17 ± 0.94	0.58 ± 0.79	—	″	0.11

Eighteen liver biopsy specimens that were within normal histological limits according to a liver histopathologist (S. E. D.) served as controls. In particular, there was no increase in the portal cell infiltrate. The clinical indication for liver biopsy in that group was investigation of asymptomatic abnormal liver enzymes. All were negative for HCV antibody; negative for HBV surface antigen; had a body mass index <30 without risk factors for insulin resistance; were negative for antimitochondrial, antinuclear, and anti–smooth muscle antibodies with normal serum immunoglobulins; had no evidence of iron overload; and had normal serum α1-antitrypsin, copper, and ceruloplasmin levels.

### Liver Immunohistochemistry

Paraffin-embedded, formalin-fixed liver tissue was cut as 5-μm sections to polylysine-coated slides. Slides were processed for immunohistochemistry as described previously.^15^ Antigen retrieval was achieved by pressure-cooking for 3 minutes in citrate buffer (pH 6.0). The following mouse monoclonal antibodies were used: anti-Mcm-2 (generated as reported previously^16^), anti-CD3, anti-CD4, anti-CD8, and anti-perforin (Novocastra, Newcastle, England). Mcm-2, a marker of cell cycle re-entry, is expressed throughout the cell cycle but not in quiescent cells. CD3 is a T lymphocyte marker. CD4 is expressed on helper T lymphocytes, and CD8 is expressed on cytotoxic T lymphocytes. Perforin expression denotes a T lymphocyte with cytotoxic potential. Biotinylated goat anti-mouse immunoglobulin was applied as a secondary antibody. Tonsil was used as a positive control, and appropriate primary antibody isotype served as a negative control on each run.

A streptavidin–horseradish peroxidase system (DAKO, Denmark) with the substrate diaminobenzidine was used to develop staining. Slides were counterstained with Harris hematoxylin, dehydrated in an ethanol series, and cleared in xylene. Cover slips were applied with DEPEX mounting medium (BDH, UK).

**Fig. 1:**
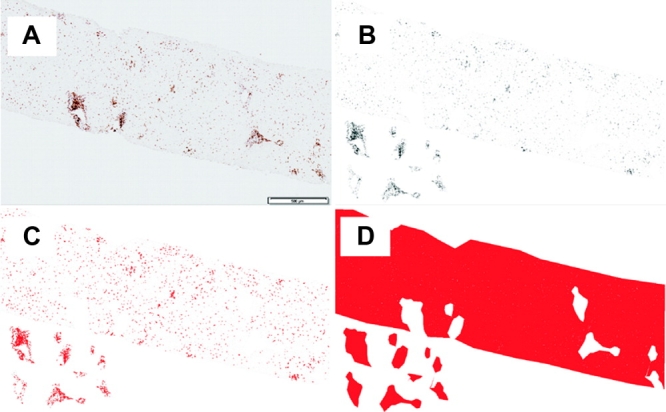
Immunohistochemical analysis using ImageJ software. (A) Representative image obtained via CD3 immunohistochemistry using the Olympus Dotslide system. The scale bar (500 μm) allows absolute areas to be calculated. (B-D) Analysis process for membranous staining. (B) ImageJ-enhanced 8-bit black and white image with portal tracts cut out to allow separate analysis of both lobular and portal regions. (C) Positive immunohistochemistry defined in red using a primary antibody-dependent standardized threshold, the area of which provides the numerator for positive immunohistochemical staining. (D) Threshold that gives a total area for both portal tract and lobular regions; the denominator. Analysis of nuclear staining is identical to membranous immunohistochemical staining, except that the immunohistochemical numerator is the number of positive cells. After a watershed is applied to separate overlapping cells, ImageJ calculates the number of positively stained cells using operator-determined shape and size characteristics.

A novel approach was used to quantify the results of immunohistochemistry in an objective fashion. A high definition image was taken at ×3.5 magnification using the Olympus Dotslide system (Olympus Microscopes, UK) (Fig. 1A). Consecutive sections were used for each antibody and the same field was selected on each occasion based on a reproducibly identifiable feature (for example, a portal tract or central vein). Immunohistochemistry was assessed using the public domain ImageJ software^17^ (National Institutes of Health, http://rsb.info.nih.gov/ij). The operator defines the scale and areas of interest, which in this series comprised the lobule and the portal tract (Fig. 1B). Images were transformed into black and white, and a threshold was established to educate the program to identify positive staining of either nuclei or membrane with each antibody specifically.

Positive nuclei are identified readily by size and shape. To separate overlapping nuclei, a watershed was applied (Fig. 1C). The results are expressed as the number of positive nuclei/mm^2^ of either lobule or portal tract.

Interpretation of membranous staining can be difficult in sections where cell density is high (leading to a number of semiquantitative and subjective scoring systems). Thus, for assessment of membrane staining the results are expressed as a proportion; in this study, the numerator was the area of cells detected as positive membranous staining by immunohistochemistry (Fig. 1C) and the denominator was the total area of interest (lobule or portal tract) (Fig. 1D). The proportion of lymphocytes positive for membrane staining for CD3, CD4, or CD8 was assessed according to either a lobular or portal distribution. Perforin staining was discrete and cytoplasmic, and the results are expressed as number of cells positive per mm^2^ of either lobule or portal tract.

### Statistics

Immunohistochemistry results were analyzed using Prism 5.0 for Windows (Graphpad, San Diego, CA). Multiple groups were analyzed with the Kruskal-Wallis test followed by Dunn's multiple comparison test. Biopsy Ishak scores were analyzed with the Mann-Whitney U test. A *P* value of less than 0.05 was regarded as significant.

## Results

### Patients

One hundred seventy-two patients positive for HCV antibody but without HCV RNA in serum (via PCR) underwent liver biopsy in our center between 1992 and 2000. One hundred two patients were excluded from the study because of evidence of a further risk factor for liver injury other than HCV exposure. Current or previous excessive alcohol intake, risk factors for insulin resistance, and concomitant liver disease, including steatohepatitis, accounted for the majority of those excluded. The remaining 70 patients were followed for a median of 7 years (range, 5–12). All patients retained anti-HCV antibody. However, during prolonged follow-up, nine of the 70 patients became HCV RNA–positive. In five patients, the result was positive on only one occasion and was determined subsequently to be a false positive reaction. However, four (5.7%) patients were confirmed repeatedly to be HCV RNA–positive and were excluded from further analysis. Detection of HCV RNA in these four cases was not associated with intercurrent illness, immune suppression, or further exposure to HCV as far as could be determined and may reflect either *de novo* infection or reactivation of previously quiescent HCV infection.

Thus, HCV exposure remained the only identified risk factor for liver injury in the remaining 66 patients, in accordance with the strict study criteria. Demographic details are described in Table 1. Seven (10.6%) patients acquired HCV through contaminated blood products, 46 (69.7%) through injecting drug use, and in the remaining 13 (19.7%) patients the source of HCV infection was undetermined. Ten (15.2%) patients had alanine aminotransferase levels that were elevated at some time during the study period, but all other laboratory parameters including alkaline phosphatase, gamma glutamyl transpeptidase, bilirubin, and platelet counts were within the normal range consistently in all patients.

### Hepatic Fibrosis and Inflammation (Figs. 2 and 3)

Only five of 66 (7.5%) patients had a normal liver biopsy; 54 of 66 (81.8%) patients had fibrosis.

**Fig. 2:**
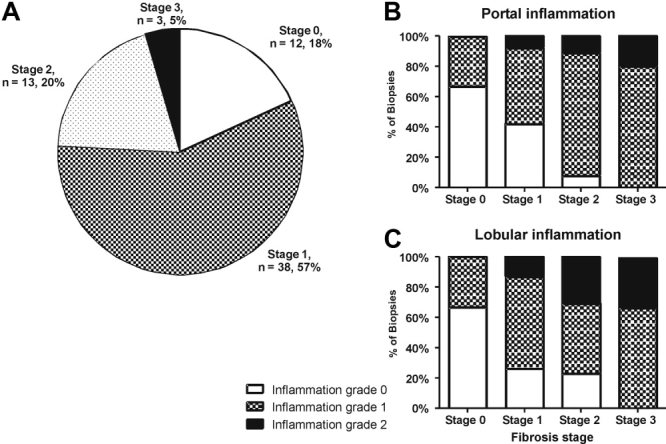
Fibrosis stage and inflammation grade in HCV antibody–positive, HCV RNA–negative subjects (n = 66). (A) Pie chart representation of fibrosis stage by modified Ishak criteria (0–6). (B) Lobular and (C) portal tract inflammation according to stage of fibrosis.

**Fig. 3:**
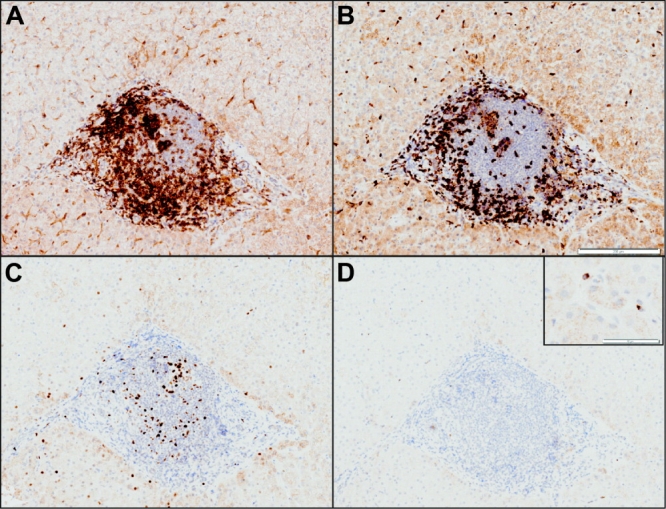
Immunohistochemistry of the portal inflammatory infiltrate from a patient with anti–HCV antibody but negative for HCV RNA. Formalin-fixed, paraffin-embedded tissue was stained for (A) CD4, (B) CD8, (C) Mcm-2, and (D) perforin. Scale bars: (B) 200 μm; (D) 50 μm (inset). Portal tracts are rich in CD3-positive cells (not shown), which are more often CD4-positive than CD8-positive. These cells express Mcm-2 and perforin rarely.

Stage 0 fibrosis was present in 12 of 66 (18.2%) patients studied; these included four (33.3%) with grade 1 portal tract inflammation and five (41.6%) with grade 1 lobular inflammation.

Stage 1 fibrosis was present in 38 patients (57.6%); 63.2% and 7.9% had grade 1 or 2 portal tract inflammation, respectively; 60.5% and 13.2% had grade 1 or 2 lobular inflammation, respectively; and 13.2% had grade 1 interface hepatitis.

Stage 2 or 3 fibrosis was present in 16 (24.2%) patients; 93.7% had grade 1 (75%) and 2 (18.7%) portal tract inflammation; 81.3% had grade 1 (56.3%) and 2 (25%) lobular inflammation; and 18.8% had grade 1 interface hepatitis.

### Bile Duct Damage and Steatohepatitis

Neither bile duct damage nor steatohepatitis were observed. Confluent necrosis was present in three biopsies (4%), never exceeding grade 1 (0–6). There was no histological evidence of covert alcohol consumption, consistent with the strict definition of the study group.

### Inflammatory Infiltrate

The inflammatory infiltrate was investigated further via immunohistochemistry in 12 nonviremic patients and compared with two control groups (see above): liver tissue from 13 viremic HCV patients matched with the nonviremic HCV antibody–positive group and 18 healthy controls. The demographic and liver biopsy characteristics of the groups are detailed in Table 2.

### Portal Tracts of Nonviremic HCV Patients Have a CD8+ Rich Infiltrate

The area of the portal tract was expanded in both groups with HCV infection when compared with healthy controls (*P* < 0.05; data not shown). There was no difference in the portal tract area between the two groups of HCV-exposed patients (whether positive or negative for HCV RNA in serum) who had been matched (intentionally) for inflammation grade.

**Fig. 4:**
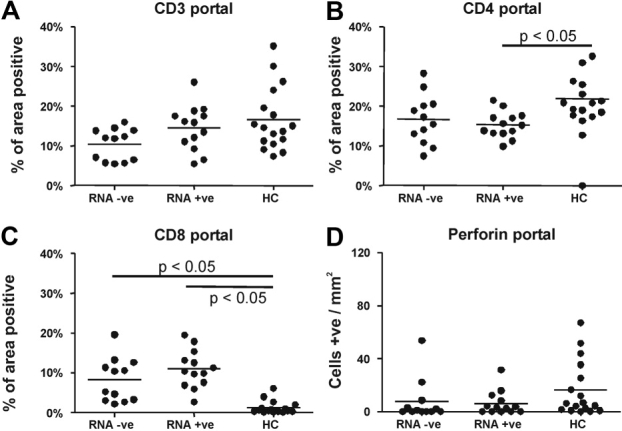
Characteristics of the portal tract infiltrate in patients with nonviremic HCV (n = 12), viremic HCV (n = 13), and healthy controls (HC) (n = 18) stained for (A) CD3, (B) CD4, (C) CD8, and (D) perforin. Results were analyzed using the Kruskal-Wallis test and Dunn's multiple comparison test.

There were no significant differences between patients and either control group regarding the area of the portal tract that expressed CD3 (Fig. 4A).

The area of the portal tract that expressed CD4 was lower in viremic patients with HCV when compared with healthy control subjects (*P* < 0.05) (Fig. 4B), but similar in both HCV-exposed groups.

The portal tract area that expressed CD8 was increased significantly in both viremic and nonviremic HCV patients when compared with healthy controls (*P* < 0.05 and *P* < 0.0001, respectively (Fig. 4C) but similar in both HCV-exposed groups. However, the number of perforin-positive cells/mm^2^ portal tract was similar in the three groups (*P* = 0.075) (Fig. 4D).

### Reduced Lobular CD3 and Perforin Expression in Nonviremic HCV Patients Compared with Healthy Controls

**Fig. 5:**
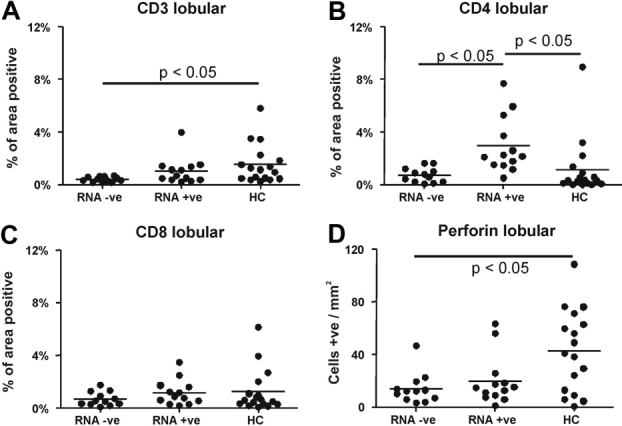
Characteristics of the lobular infiltrate in patients with nonviremic HCV (n = 12), viremic HCV (n = 13), and healthy controls (HC) (n = 18). Biopsies were stained for (A) CD3, (B) CD4, (C) CD8, and (D) perforin. Results were analyzed using the Kruskal-Wallis test and Dunn's multiple comparison test.

The proportion of the lobular area positive for CD3 in both viremic and nonviremic patients was reduced compared with healthy control subjects (*P* = 0.0132) (Fig. 5A), but was similar in both HCV-exposed groups.

There was an increase in the lobular area that expressed CD4 in viremic patients (median, 2.13%; interquartile range, 1.49–4.49) compared with both nonviremic patients (median, 0.68%; interquartile range, 0.22–1.16) (*P* < 0.05) and healthy controls (median, 0.32%; interquartile range, 0.10–1.13) (*P* < 0.05) (Fig. 5B). However, review of the staining pattern for CD4 in liver tissue revealed that most of the signal localized to sinusoidal lining cells, with the effect most marked in patients with viremia (Supplementary Fig. 1A). CD4 expression had a similar pattern but was less marked in nonviremic patients (Supplementary Fig. 1B) and was rare in healthy controls. CD4 lymphocytes were detected rarely in both study groups and when identified were sinusoidal.

There were no differences between the three study groups in terms of the lobular area that expressed CD8 (*P* = 0.477) (Fig. 5C). However, perforin expression was reduced in both viremic and nonviremic HCV patients compared with healthy controls, but similar in both HCV-exposed groups (*P* = 0.0314) (Fig. 5D).

### Portal Tract Lymphocytes in HCV Infection Are Mcm-2–Negative Independent of Viremia

Portal tract cells in both viremic and nonviremic HCV patients had minimal expression of mcm-2 (Fig. 3C); expression in both groups was reduced significantly compared with healthy controls (*P* = 0.0004) (Supplementary Fig. 2A).

### Increased Lobular Expression of Mcm-2 in Viremic and Nonviremic HCV Patients

Nonviremic patients had significantly greater expression of Mcm-2 within lobular areas compared with healthy controls (*P* = 0.0005) (Supplementary Fig. 2B). This was almost exclusively confined to hepatocytes and infiltrating inflammatory cells were always negative. There were no differences between the hepatocyte expression of Mcm-2 between viremic and nonviremic HCV-positive patients as described.^18^

## Discussion

HCV infection leads to chronic viremia in the majority of individuals exposed to HCV. The natural history in this group, the risk factors for progressive injury and the benefits of antiviral therapy are well established. However, the clinical status of the minority without viremia after exposure to HCV is less clear. It is uncertain whether this group has resolved infection, with or without long-term immunity and protection from further exposure to HCV or, alternatively, low-level viral replication, where HCV RNA can only be detected within the liver.^11^,^19^ Neither the natural history nor the liver histology in this cohort has been described in detail.

We followed a cohort of HCV-exposed patients without viremia at presentation for a median of 7 years, many of whom were identified at a time when there was uncertainty regarding the significance of a failure to detect HCV RNA at first assessment. With the aid of liver biopsy in all of these patients and critically, careful subsequent exclusion of all patients with a possible alternative cause of chronic liver disease, we have been able to challenge the view that nonviremic HCV-exposed patients have resolved infection. First, viremia was detected eventually in 5.7% of this group, a proportion that may increase with time; second, just 7.5% of patients had normal histology; third, 92% of patients had inflammation within the liver, while 82% had fibrosis, which in about a quarter would have been sufficient to prompt consideration of antiviral therapy if the patients had been viremic; finally, when cases without viremia were compared with viremic patients matched for grade of inflammation and stage of fibrosis, the phenotype of the inflammatory infiltrate was similar and distinct from that in healthy controls.

These data are consistent with the hypothesis that nonviremic patients exposed to HCV have chronic low-level, probably hepatic viral replication that is associated with a lower risk of progressive liver injury compared with viremic patients. There are other possibile explanations for the histological abnormalities, including as yet unknown viral infections or nonalcoholic fatty liver disease without histological features of steatohepatitis.

Serum from 80 HCV RNA–negative patients was subjected to ultracentrifugation before repeating the assay for HCV RNA; HCV RNA was still not detected (Rolfe K and Curran MD, personal communication). The findings are thus consistent with several studies that have described the detection of HCV RNA in liver tissue in nonviremic HCV-exposed individuals.^11^,^19^–^21^ This view would also be consistent with a failure to demonstrate sterilizing immunity against HCV in humans or primates,^22^ and it is possible that HCV is a lifelong infection in many more cases than has been supposed hitherto. Perhaps the most important question to address in this cohort is why such cases have lower levels of viral replication. The long-term histology in those treated successfully with pegylated interferon-α and ribavirin will be of interest in this context, because loss of the inflammatory infiltrate would be consistent with eradication of HCV, while ongoing inflammation, as in this series, would be indicative of low-level HCV replication.

Inflammation in the liver is a sensitive indication of hepatic disorder, but indirect evidence of infection. The best evidence of infection in nonviremic HCV-exposed patients would be the demonstration of HCV genomic material and replicative intermediates in the livers of such cases. Both positive- and negative-strand HCV RNA have been identified in the liver tissue of nonviremic HCV patients with normal alanine aminotransferase values^11^; that study also demonstrated that HCV RNA was present in serum after ultracentrifugation.^20^ This suggests nonviremic HCV patients are defined by insensitive tests. In a series of patients from the same authors with HCV RNA present in liver but without viremia, 15% had fibrosis, including 4% with cirrhosis.^13^ This contrasts with 82% with some degree of fibrosis in our series, a difference that may be explained by the longer duration of follow-up in this series compared with that of the Spanish group.^11^

Immunohistochemical analysis was revealing. There were consistent differences between HCV-exposed cases (irrespective of viremic status) and healthy controls; in contrast, no differences were detected between viremic and nonviremic HCV-exposed patients matched for inflammation for any other parameter. Thus, the portal tracts were expanded with nonproliferating (Mcm-2–negative) T cells enriched with CD8+ T cells and depleted of CD4+ T cells in HCV-exposed patients relative to healthy controls. However, the proportion of cells expressing perforin, a marker of cytotoxic potential, was low and similar in all three groups.^23^ The lobular infiltrate was CD3+ T cell–depleted and perforin-negative in both HCV-exposed groups relative to healthy controls.

Mcm-2 expression, a marker of cell cycle entry, was increased in hepatocytes in both HCV study groups. A previous study indicated that hepatocytes in HCV-exposed patients had evidence of cell cycle entry without cell cycle progression—a state of cell cycle arrest—that correlated with fibrosis stage.^18^ Many viruses replicate more efficiently in cell cycle–arrested host cells,^24^ and Mcm-2–positive hepatocytes may be either HCV-infected or regenerating in response to ongoing liver injury. In either case, the finding is indicative of an ongoing liver insult in both viremic and nonviremic HCV-exposed groups.

Stringent selection of nonviremic HCV-exposed patients with HCV as the only risk factor for liver injury revealed abnormal liver histology in almost all cases. How should such cases be managed? For now, it might be wise to continue to follow such cases to determine whether HCV RNA will be detected eventually and to determine the natural history in this cohort. In the future, testing for HCV in serum or tissue may improve, and the proportion of HCV RNA–negative patients may fall. Intervention with antiviral therapy cannot be justified based on our current knowledge of the natural history; however, it will be intriguing to determine the late histology in HCV RNA–positive cases treated successfully to see whether these revert to normal histology or something more akin to the findings in the nonviremic group in this series. However, a possible role for HCV in nonviremic patients with a second risk factor for liver injury does need to be addressed, and it is possible that the threshold for investigating such cases more thoroughly will be reduced.

Whether this group is analogous to patients with occult HBV infection^25^ who can experience reactivation of viral replication in the face of profound immunosuppression^26^ is not known. Previous studies comparing rates of HBV and HCV reactivation suggest that it is much less common with HCV and indeed may not occur; in a study of 305 patients receiving corticosteroid containing chemotherapy for hematological malignancy, there were nine reactivations of HBV infection but no reactivation of HCV viremia, despite a four-fold higher prevalence of nonviremic HCV than HBV.^27^ A more analogous situation may be the outcome of antiviral therapy, where a small proportion of individuals eventually become HCV RNA–positive despite sustained virological response (SVR). In one study of individuals who achieved SVR after previously failing an initial course of antiviral therapy, the viral recurrence rate after SVR was 11.3%.^28^ Furthermore, viral RNA can be detected in peripheral blood lymphocytes and macrophages from those individuals who have successfully achieved SVR.^29^

An unexpected but consistent observation was that CD4 expression in the lobule was prominent in sinusoidal lining cells in HCV-exposed patients. The pattern was most consistent with endothelial expression, and expression was most marked in viremic patients. The majority of CD4 staining was sinusoidal, which caused difficulty with the semiautomated count of lymphocytes that were therefore assessed via more conventional means. The significance of sinusoidal lining cell CD4 expression will be pursued in a separate study. CD4 staining has been demonstrated in both glomerular and brain endothelial tissue in HIV-1 infection.^30^,^31^ In the latter study, brain endothelial cells expressed both CD4 and chemokine receptors, suggesting a permissive role in HIV infection.^30^

In conclusion, we have identified a cohort of individuals with no risk factor for liver injury other than previous HCV exposure. We have demonstrated that these patients with nonviremic HCV have a CD8+ rich hepatic inflammatory infiltrate and the great majority have evidence of hepatic fibrosis.

## Abbreviations

HBV: hepatitis B virus
HCV: hepatitis C virus
HIV: human immunodeficiency virus
PCR: polymerase chain reaction
SVR: sustained virological response
