# Supplementary figures and images for: Histological changes in HCV antibody–positive, HCV RNA–negative subjects suggest persistent virus infection
Source: Hepatology. 2008 Dec;48(6):1737–45. doi: 10.1002/hep.22484 (PMC2680218; doi:10.1002/hep.22484)

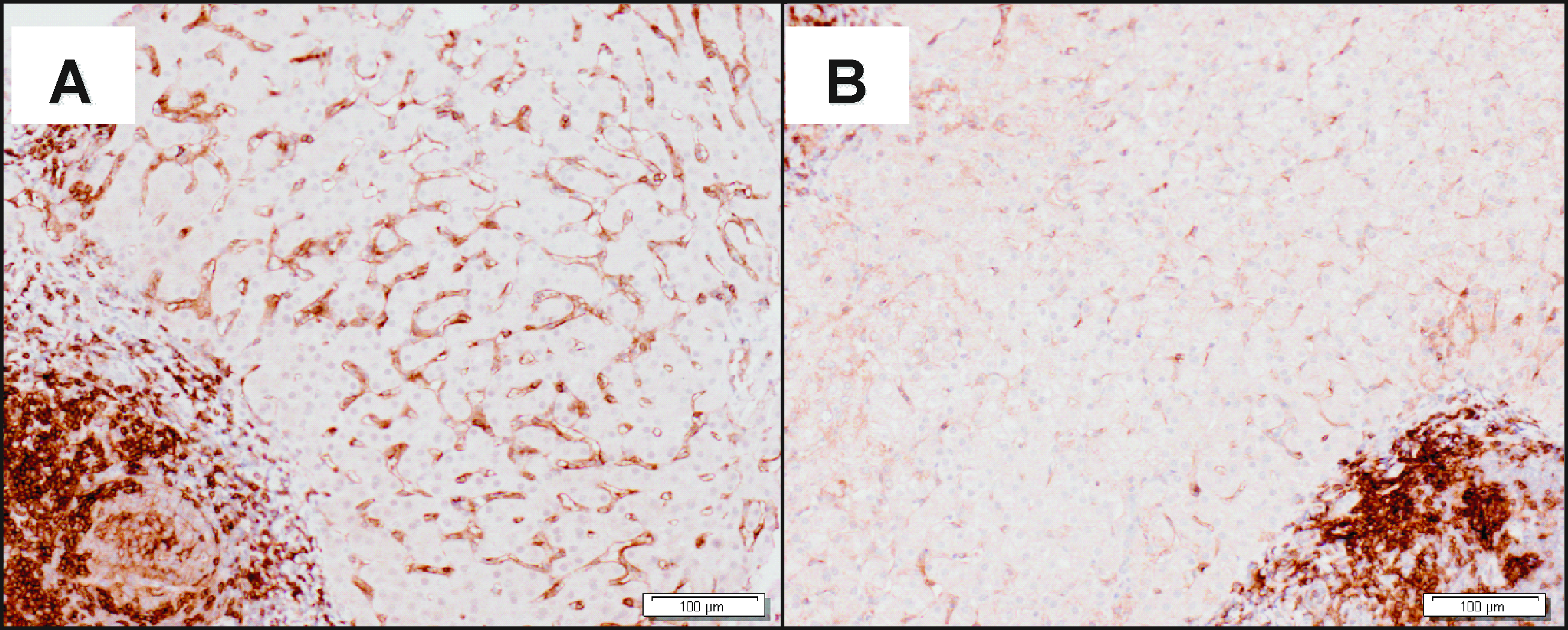

Supplement: Supplementary file 1 [file hep0048-1737-SD1.tif]

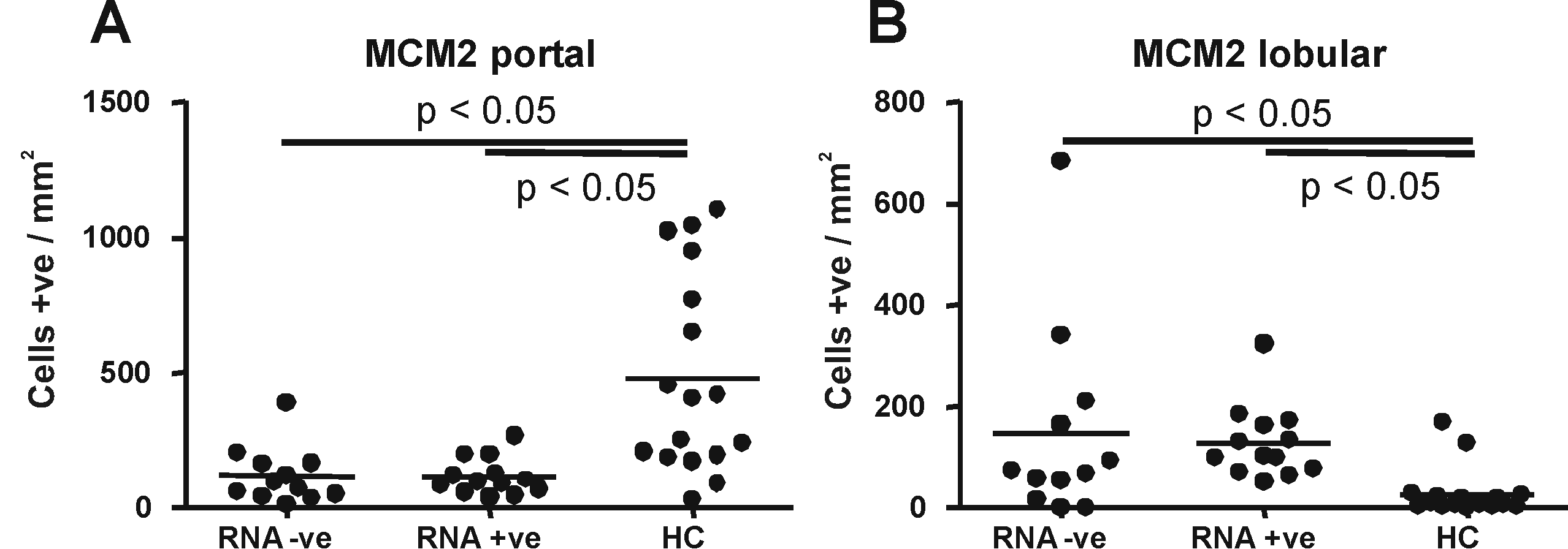

Supplement: Supplementary file 2 [file hep0048-1737-SD2.tif]
